# A Prebiotic Diet Alters the Fecal Microbiome and Improves Sleep in Response to Sleep Disruption in Rats

**DOI:** 10.3389/fnins.2022.889211

**Published:** 2022-05-24

**Authors:** Samuel J. Bowers, Keith C. Summa, Robert S. Thompson, Antonio González, Fernando Vargas, Christopher Olker, Peng Jiang, Christopher A. Lowry, Pieter C. Dorrestein, Rob Knight, Kenneth P. Wright, Monika Fleshner, Fred W. Turek, Martha H. Vitaterna

**Affiliations:** ^1^Center for Sleep and Circadian Biology, Northwestern University, Evanston, IL, United States; ^2^Department of Neurobiology, Northwestern University Weinberg College of Arts and Sciences, Evanston, IL, United States; ^3^Division of Gastroenterology & Hepatology, Department of Medicine, Northwestern University Feinberg School of Medicine, Chicago, IL, United States; ^4^Department of Integrative Physiology, University of Colorado, Boulder, Boulder, CO, United States; ^5^Center for Neuroscience, University of Colorado, Boulder, Boulder, CO, United States; ^6^Department of Pediatrics, University of California, San Diego School of Medicine, La Jolla, CA, United States; ^7^Collaborative Mass Spectrometry Innovation Center, Skaggs School of Pharmacy & Pharmaceutical Sciences, University of California, San Diego, La Jolla, CA, United States; ^8^Center for Microbiome Innovation, University of California, San Diego, La Jolla, CA, United States; ^9^Department of Computer Science and Engineering, University of California, San Diego, La Jolla, CA, United States; ^10^Department of Bioengineering, University of California, San Diego, La Jolla, CA, United States; ^11^Sleep and Chronobiology Laboratory, University of Colorado, Boulder, Boulder, CO, United States; ^12^The Ken & Ruth Davee Department of Neurology, Northwestern University Feinberg School of Medicine, Chicago, IL, United States; ^13^Department of Psychiatry and Behavioral Sciences, Northwestern University Feinberg School of Medicine, Chicago, IL, United States

**Keywords:** sleep, sleep restriction, prebiotic, microbiome, microbiome-gut-brain axis

## Abstract

Sleep disruption is a challenging and exceedingly common physiological state that contributes to a wide range of biochemical and molecular perturbations and has been linked to numerous adverse health outcomes. Modern society exerts significant pressure on the sleep/wake cycle *via* myriad factors, including exposure to electric light, psychological stressors, technological interconnection, jet travel, shift work, and widespread use of sleep-affecting compounds. Interestingly, recent research has identified a link between the microbiome and the regulation of sleep, suggesting that interventions targeting the microbiome may offer unique therapeutic approaches to challenges posed by sleep disruption. In this study, we test the hypothesis that administration of a prebiotic diet containing galactooligosaccharides (GOS) and polydextrose (PDX) in adult male rats improves sleep in response to repeated sleep disruption and during recovery sleep. We found that animals fed the GOS/PDX prebiotic diet for 4 weeks exhibit increased non-rapid eye movement (NREM) and rapid eye movement (REM) sleep during 5 days of sleep disruption and increased total sleep time during 24 h of recovery from sleep disruption compared to animals fed a control diet, despite similar baseline sleep characteristics. Further, the GOS/PDX prebiotic diet led to significant changes in the fecal microbiome. Consistent with previous reports, the prebiotic diet increased the relative abundance of the species *Parabacteroides distasonis*, which positively correlated with sleep parameters during recovery sleep. Taken together, these findings suggest that the GOS/PDX prebiotic diet may offer an approach to improve resilience to the physiologic challenge of sleep disruption, in part through impacts on the microbiome.

## Introduction

Sleep disruption is a common problem in modern society. Sleep deprivation increases the risk of motor vehicle accidents (Barger et al., 2005; Bioulac et al., 2017), workplace injuries (Nakata, 2011), and medical errors (Trockel et al., 2020). Chronic sleep disruption has been associated with many adverse health consequences, including, but not limited to, increased rates of cardiovascular, metabolic, gastrointestinal, neurological, and psychiatric diseases (Kecklund and Axelsson, 2016; Liew and Aung, 2021). Many aspects of the modern environment contribute to sleep disruption: electric light, screen exposure, technological interconnection, societal and workplace expectations for near constant availability, jet travel, shift work, and widespread use of sleep-interfering chemicals such as caffeine (Grandner, 2017). Although lifestyle modifications may mitigate some of these factors, these are not often effective under all conditions nor are they widely adopted or sustainable over long periods of time. Furthermore, it seems unlikely that societal, environmental, and cultural factors contributing to insufficient sleep duration and poor sleep habits will reverse. Thus, interventions aimed at improving resilience to insufficient sleep may offer a viable strategy for mitigating adverse consequences.

Interestingly, recent work has demonstrated bidirectional connections between sleep and the microbiome in rodent models (Thompson et al., 2016, 2020; Bowers et al., 2020; Wang et al., 2022) and humans (Matenchuk et al., 2020). Considering the context of the large and growing bodies of literature linking adverse physiologic consequences and multiple diseases to sleep disruption (Kecklund and Axelsson, 2016; Liew and Aung, 2021) and to changes to the intestinal microbiome, or dysbiosis (Battson et al., 2018; Halverson and Alagiakrishnan, 2020; Meng et al., 2020), strategies targeting the structure and function of the microbiome are an exciting potential therapeutic opportunity (Wolter et al., 2021). One such approach is *via* dietary supplementation with prebiotics, which are compounds neither absorbed nor actively metabolized by human hosts but are selective substrates for intestinal bacteria thought to be beneficial (Manning and Gibson, 2004). Galactooligosaccharides (GOS) and polydextrose (PDX) are examples of prebiotics that have been shown to impact physiological processes in different model systems (Macfarlane et al., 2008; Do Carmo et al., 2016), including a recent report showing that GOS/PDX supplementation in rats accelerates recovery in response to environmental disruption of circadian rhythms (Thompson et al., 2021).

In this study, we test the hypothesis that dietary supplementation with the prebiotics GOS and PDX improves sleep in response to sleep deprivation and recovery sleep in adult male rats. We demonstrate that prebiotic diet supplementation led to changes in the structure and predicted function of the microbiome. The prebiotic diet did not impact baseline sleep, yet, surprisingly, promoted increased non-rapid eye movement (NREM) and rapid eye movement (REM) sleep during the sleep deprivation protocol as well as increased total sleep during recovery. These sleep changes were positively correlated with the relative abundance of the bacterium *Parabacteroides distasonis*, which we and others have previously shown to be increased by GOS/PDX supplementation and which may promote resilience in the setting of physiologic challenges such as chronic disruption of circadian rhythms (Thompson et al., 2021). An analysis of fecal bile acids demonstrated associations with *P. distasonis* and other bacteria impacted by the prebiotic diet, suggesting a potential mechanism by which the prebiotic diet may exert physiologic effects. These findings indicate that dietary interventions targeting the microbiome may provide resilience in the context of physiologically challenging environmental stimuli such as insufficient sleep.

## Materials and Methods

### Animals and Experimental Design

**FIGURE 1:**
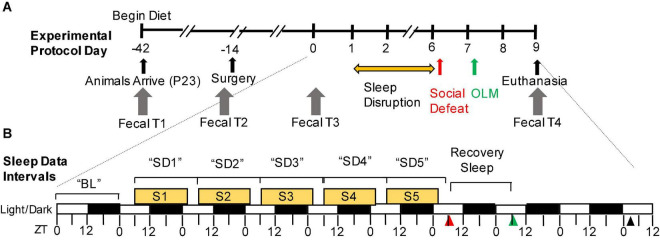
Experimental timeline. **(A)** Overview of the experimental protocol. Experimental protocol days are depicted along the axis of the timeline. The yellow horizontal arrow denotes the sleep disruption portion of the protocol. The red and green arrows denote social defeat and object learning memory, respectively. Days of fecal sample collection are indicated with gray arrows. **(B)** Detail of indicated period that includes the sleep disruption protocol, social defeat stress (red arrowhead), object location memory (green arrowhead), and terminal sample collection (black arrowhead). Tick marks along the bottom of the timeline axis indicate Zeitgeber time (ZT) on that day, white/black rectangles indicate 12-h light/dark phases, and yellow rectangles indicate time periods of sleep disruption for the sleep-disrupted groups (ZT6-ZT2). EEG/EMG recording intervals, as used in the forthcoming figures, are indicated above the timeline. BL, baseline; OLM, object location memory; P, postnatal day; S, 20-h sleep disruption window; SD, 24-h sleep disruption day; ZT, Zeitgeber time.

Figure 1 presents a schematic of the experimental protocol. Twelve cohorts of eight 23-day old male Sprague Dawley rats (Envigo Laboratories, Madison, Wi, United States) were used for this experiment (*N* = 96). A total of 13 animals did not complete the experimental protocol and were thus eliminated from all analyses, for a total *N* = 83. No explicit power analysis was used, the sample size was selected to ensure an adequate number of biological replicates for the primary outcome measure: polysomnographic sleep recording (target of *N* = 10–12/experimental group). The experiment consisted of eight experimental groups in a 2 × 2 × 2 design (control diet *vs* prebiotic diet, *ad* libitum sleep *vs* sleep disruption, no social defeat *vs* social defeat). Rats were pair-housed until electroencephalographic (EEG) and electromyographic (EMG) implant surgery at 7 weeks of age, after which they were individually housed until the end of the experiment.

After placement of rats in cages on the day of arrival, diet groups (control diet *vs* prebiotic diet) were randomly assigned to the different cages. After EEG/EMG surgery, rats were assigned to further experimental groups (*ad* libitum sleep *vs* sleep disruption and no social defeat *vs* social defeat) randomly, with an effort to ensure prior cagemates were in different groups. Male Long Evans rats (Envigo Laboratories) were used as aggressors in the social defeat model (see below). All rats were maintained on a 12:12 light:dark cycle at room temperature (23 ± 2°C) with food and water available *ad* libitum throughout the experiment. All protocols were approved in advance by the Northwestern University Institutional Animal Care and Use Committee. Zeitgeber time (Zt) is defined as the number of hours after the onset of the light period (light onset = Zt0).

### Experimental Diets

Upon arrival to the facility, rats were started on *ad libitum* control or prebiotic diets as previously described (Thompson et al., 2021). The control diet was Envigo Teklad diet TD.110883 (Envigo Teklad, Madison, WI, United States). The prebiotic diet consisted of the control diet supplemented with galactooligosaccharides [GOS, 21.23 total g/kg (7.00 active g/kg); FrieslandCampina, Zwolle, Netherlands] and polydextrose (PDX, 7.69 total g/kg (7.00 active g/kg); Danisco, Terre Haute, IN, United States). The prebiotic diet was custom-made by Envigo Teklad (TD.110889). The control and prebiotic diets are isocaloric and contain similar macronutrient, vitamin, and mineral levels, as previously described (Thompson et al., 2016, 2021).

### EEG/EMG Implantation Surgery

Four weeks after arrival, and 14 days prior to baseline sleep (i.e., on day –14), rats were implanted with electroencephalographic/electromyographic (EEG/EMG) sleep recording devices (Pinnacle Technologies, Lawrence, KS, United States). Surgical procedures were performed using a rat stereotaxic apparatus with standard aseptic techniques in a ventilated, specially equipped surgical suite. Anesthesia was induced by isoflurane gas. The EEG/EMG headmount consisted of a plastic 6-pin connector attached to four EEG electrodes and two EMG electrodes. Four stainless steel screws serving as two EEG leads and grounds were screwed into the skull with one lead located 5 mm anterior to bregma and 2 mm lateral to the central suture, another 1 mm anterior to bregma, 2 mm lateral to the central suture, and the other two at 1 mm anterior to lambda and 2.5 mm lateral to each side of the central suture. The exposed ends of two stainless steel Teflon-coated wires serving as EMG leads were then inserted into the nuchal muscles using a pair of forceps. The entire headmount was then sealed by dental acrylic and, at the front and the back of the implant, sutures were used to close the incision. Indirect heat support was provided until recovery from anesthetic by placing a heating pad underneath half of the cage the animals were returned to after surgery. Subcutaneous injection of analgesic meloxicam (2 mg/kg; Norbrook Laboratories, Northern Ireland) was given to the animals at the time of surgery and once more on the following day.

### Sleep Recording and Analysis

After surgery, rats were moved into cylindrical sleep recording cages (Pinnacle Technologies) within individual acoustically-isolated and Faraday-shielded chambers. Two days before baseline sleep, the headmount was connected to the transmission tether. Cages had corncob bedding as well as food and water available *ad libitum*. Sleep was recorded for a 24-h baseline, then recordings were begun at the start of sleep restriction protocol (ZT6), in which rats were permitted 4 h of interrupted sleep opportunity per 24 h. At the end of the fifth 20-h sleep disruption session, rats were unplugged from their EEG/EMG tethers for social defeat (see below). Upon return to home cages, sleep recording resumed for 24 h (ZT7-ZT7) until rats were unplugged from their EEG/EMG tethers for evaluation in the object location memory (OLM) test. Data were collected using Pinnacle Acquisition software (Pinnacle Technologies), then scored as non-rapid eye movement sleep (NREM), rapid eye movement sleep (REM), or wake in 10 s epochs using machine learning-assisted sleep scoring program as described previously (Gao et al., 2016).

The initiation of a bout of NREM, REM, or wake was defined by the occurrence of two consecutive epochs of NREM, REM, or wake (respectively). A bout was terminated when two consecutive epochs failed to match the state of that bout. For example, a NREM sleep bout was initiated by two consecutive NREM epochs and was terminated when two consecutive non-NREM epochs occurred. A brief arousal was defined as a single epoch of wake within a sleep bout. The delta power band was defined as 0.5–4 Hz, theta as 4–8 Hz, alpha as 8–11 Hz, sigma as 11–15 Hz, and beta as 15–30 Hz. Relative power was calculated as the raw power (μV^2^) in a particular band divided by the total power in all bands.

### Sleep Disruption Protocol

After baseline sleep recordings, half of the rats were tested in the sleep disruption protocol. Sleep disruption was achieved using a commercially available system integrated into the chambers (Pinnacle Technologies), which simulates the gentle handling technique *via* a rotating metal bar (22 cm in length) attached to a post at the center of the cage. For sleep disruption days, the rotation speed of the bar was set at seven rotations per minute with reversals of rotation direction (i.e., clockwise *vs.* counterclockwise) set to occur at semi-random intervals of 10 ± 10 s. The bar was programmed to rotate for 20 h per day (ZT6-ZT2) and was stationary for 4 h per day (ZT2-ZT6), for 5 days total. Experimenters visually inspected rats at regular intervals during the sleep disruption windows to ensure that the bar mechanism was functioning properly and that the sleep-disrupted rats were awake. Control animals were placed in identical cages with bars that remained stationary throughout the experiment.

### Social Defeat Protocol

Male Long Evans rats were singly housed in large (44 cm × 24 cm × 21 cm) polycarbonate cages and screened for aggressive behavior before the experiment. Rats that began to injure their opponents by harmful bites during screening were not used for the social defeat procedure. On the day of the acute social defeat stress exposure, half of the experimental rats were introduced into the cage of an aggressor (testing done at ∼ZT6). As soon as the aggressor rat attacked and defeated the intruder rat, the intruder was covered with a 25 cm × 15 cm × 15 cm metal mesh cage while still inside the aggressor cage and left in place for 1 h. Control animals not receiving social defeat were unplugged from the recording tether and placed in a clean cage in a quiet room.

### Object Location Memory Task

The object location memory (OLM) task is a hippocampal-dependent memory task (Murai et al., 2007) that is sensitive to stress exposure (Cazakoff et al., 2010; Howland and Cazakoff, 2010). All rats underwent testing in the OLM task the day after social defeat, beginning at ZT7. Rats were placed in a dimly lit (∼50 lux) 53 cm × 53 cm × 30 cm arena with no objects and allowed to explore for 5 min. Approximately 30–40 min later, they were returned to the chamber, this time containing two identical cylindrical objects (100 mL pyrex bottles with caps) on the same side of the arena. Rats were allowed to explore the arena for 5 min and after 90 min in their home cage were allowed to explore the arena again, with one object moved. Exploration of the moved object for longer than the non-moved object is considered evidence of successful acquisition of contextual memory (Murai et al., 2007). This is denoted by a “location index” expressed as a percentage of time [100 × (time exploring moved object/total time exploring either object)], with values significantly greater than 50% representing evidence of retained contextual memory (Ennaceur et al., 1997). In this experiment, location index was quantified during the 5 min of the testing session. LimeLight (Actimetrics, Wilmette, IL, United States) behavioral software was used to track the path of locomotor activity of each animal within the open field over time. De-identified video files were scored by two experimenters and average location indices were reported.

### Fecal Sample Collection

Fecal samples were collected at four timepoints: (T1) the day of arrival; (T2) one to 2 days before surgery (4 weeks on diet); (T3) at baseline sleep (6 weeks on diet); and (T4) at the end of the experiment (see Figure 1A). Each collection occurred on days where clean cages were provided, so rats were placed into a clean chamber with fresh bedding and food and monitored closely until at least two fresh fecal pellets from each cage were collected. Only spontaneously voided pellets were collected. Samples were placed into individual 1.5 mL microfuge tubes, and frozen at −80°C until microbiome and metabolome analysis, at which point one sample from each animal (or two from each cage for timepoints T1 and T2) was cut in half. One half was used for fecal microbiome analysis and the other half was used for fecal metabolome analysis. At each collection timepoint, duplicate samples of bedding, water, food, and blank tubes were also collected.

### Microbiome Analysis

To evaluate the impact of the prebiotic diet on the microbiome and to assess for correlations between our primary outcome of interest, the response to sleep disruption, and changes to the microbiome, 16S rRNA gene sequencing was used on a total of 334 fecal and 63 environmental samples. DNA was extracted from fecal samples and the V4 region of the 16S rRNA gene was amplified using the 515f/806rB primer pair with the barcode on the forward read (Apprill et al., 2015) and sequenced as previously described (Caporaso et al., 2012) using an Illumina MiSeq. Sequence data were processed using Deblur v1.1.0 (Amir et al., 2017), trimming to 150 nucleotides to create sub-operational-taxonomic-units (sOTUs). These were then inserted into the Greengenes 13_8 (Mcdonald et al., 2012) 99% reference tree using SATe-enabled Phylogenetic Placement (SEPP) (Mirarab et al., 2012). SEPP uses a simultaneous alignment and tree estimation strategy (Liu et al., 2009) to identify placements for sequence fragments within an existing phylogeny and alignment. Taxonomy was assigned using an implementation of the Ribosomal Database Project (RDP) classifier (Wang et al., 2007) as implemented in QIIME2 (Caporaso et al., 2010). Microbiome data were generally analyzed using the Qiita (Gonzalez et al., 2018) and Quantitative Insights Into Microbial Ecology 2 (QIIME2, version 2018.4) bioinformatics software packages (Caporaso et al., 2010; Bolyen et al., 2019).

Microbial diversity analysis was performed at a rarefied depth of 9,000 reads, resulting in the removal of 21 fecal samples that did not have 9,000 reads. Beta diversity, which measures microbial similarity and dissimilarity between populations of samples, was assessed using weighted and unweighted UniFrac distance matrices as previously described (Lozupone et al., 2011). These matrices were used to generate principal coordinate analysis (PCoA) plots and to perform permutational multivariate analysis of variance (PERMANOVA) in QIIME2. Alpha diversity, which measures microbial taxonomic richness and evenness within a single sample, was calculated using scikit-bio 0.5.1 as implemented by QIIME2. Relative differential abundance was assessed at the OTU, genus, and species levels using analysis of the composition of microbiomes (ANCOM) (Mandal et al., 2015) as implemented in QIIME2. Count numbers of a taxon of interest were centered log ratio transformed in order to perform correlational analysis with physiological variables (Gloor et al., 2017).

### PICRUSt2 Analysis of 16S rRNA Gene Data

We inferred the microbial gene content from the taxa abundance using the software package Phylogenetic Investigation of Communities by Reconstruction of Unobserved States (PICRUSt2^1^; v2.1.4-b) (Langille et al., 2013). This tool allows assessment of functional capacity of a microbiome using 16S rRNA gene sequencing data. To identify differentially abundant functional pathways and enzymes, DESeq2 (version 1.14.1) was performed using the Bioconductor R package in RStudio (version 1.2.1335, RStudio Inc.).

### Metabolome Analysis

A total of 334 fecal and 63 environmental samples were processed for fecal metabolome analyses. A clean stainless-steel bead (Qiagen Catalog# 69989) and 1.5 mL chilled extraction solvent (50% MeOH) was added to each sample. The samples were then homogenized for five min at 25 Hz using a TissueLyser II system (Qiagen Catalog# 85300) and allowed to incubate for 20 min at −20°C. The fecal homogenates were then centrifuged at 14,000 rpm for 15 min at 4°C. 1.2 mL aliquots were then transferred into Nunc 2.0 mL DeepWell plate (Thermo Catalog# 278743) and frozen at −80 °C prior to lyophilization using a FreeZone 4.5 L Benchtop Freeze Dryer with Centrivap Concentrator (Labconco). Wells were resuspended with 200 μL of resuspension solvent (50% MeOH spiked with 2.0 μM sulfadimethoxine), vortexed for 30 s, and centrifuged at 2,000 rpm for 15 min at 4°C. 150 μL of the supernatant was transferred into a 96-well plate and maintained at 4°C prior to LC-MS analysis. A resuspension solvent QC and a six standard mix QC (50% MeOH spiked with 1.0 μM sulfamethazine, 1.0 μM sulfamethizole, 1.0 μM sulfachloropyridazine, 1.0 μM amitriptyline, and 1.0 μM coumarin 314) was run every 12th sample to assess sample background, carry over, chromatography behavior, peak picking, and plate effects.

Fecal extracts were analyzed using an ultra-high performance liquid chromatography system (Vanquish, Thermo) coupled to a hybrid quadrupole-Orbitrap mass spectrometer (Q-Exactive, Thermo) fitted with a HESI probe. Reverse phase chromatographic separation was achieved using a Kinetex C18 1.7 μm, 100 Å, 50 × 2.1 mm column (Phenomenex) held at 40 °C with a flow rate of 0.5 mL/min. 5.0 μL aliquots were injected per sample/QC. The mobile phase used was (A) 0.1% formic acid in water and (B) 0.1% formic acid in acetonitrile. The elution gradient was: 5.0% B for 1 min, increased to 100% B in the next 8 min, held at 100% B for 2 min, returned to 5.0% B in 0.5 min, equilibrated at 5.0% B for 2 min. Positive electrospray ionization parameters were: sheath gas flow rate of 52 (arb. units), aux gas flow rate of 14 (arb. units), sweep gas flow rate of 3 (arb. units), spray voltage of 3.5 kV, capillary temperature of 270 °C, S-Lens RF level of 50 (arb. units), and aux gas heater temperature of 435 °C. Negative electrospray ionization parameters were: sheath gas flow rate of 52 (arb. units), aux gas flow rate of 14 (arb. units), sweep gas flow rate of 3 (arb. units), spray voltage of 2.5 kV, capillary temperature of 270 °C, S-Lens RF level of 50 (arb. units), and aux gas heater temperature of 435 °C. MS data were acquired using a data dependent acquisition method with a resolution of 35,000 in MS^1^ and 17,000 in MS^2^. An MS^1^ scan from 100–1,500 *m/z* was followed by an MS^2^ scan, produced by collision induced disassociation, of the five most abundant ions from the prior MS^1^ scan.

The orbitrap files (.raw) were exported to mzXML files using MSConvert (Chambers et al., 2012). Feature detection of the MS^1^ data was performed using MZmine2 (Pluskal et al., 2010).

The resultant feature tables contained 12,570 features (fecal). Feature tables were also generated for samples of the control and prebiotic diets, containing 2,379 features. All these features were removed from the fecal feature table, resulting in a table of 10,229 non-dietary fecal metabolites. To annotate features with a metabolome standard initiative (MSI) level 1 level of confidence, mass and retention time were aligned and MS/MS fragmentation pattern was compared between features and 20 purified bile acid reference standards. Annotated features were normalized to an internal standard followed by a row sum (total bile acid ion count) normalization.

### Statistical Analyses and Software

All graphs depict the mean ± SEM unless otherwise stated. Initial analysis of sleep data used in this study revealed that while 8/8 variables passed heteroscedasticity testing, only 4/8 passed multiple normality tests. Therefore, we elected to use either non-parametric testing or statistical approaches like mixed effects modeling that have been shown to be fairly robust in the setting of mild-moderate violations of assumptions (Schielzeth et al., 2020; Knief and Forstmeier, 2021). All PCoA plots (Supplementary Figure 1) were generated using the EMPeror visualization tool as implemented in QIIME2 (Vazquez-Baeza et al., 2013). Microbiome data processing and analysis, including microbiome PERMANOVA, were performed in QIIME2 as outlined above. Wilcoxon Rank-Sum tests and linear mixed effect modeling of metabolome data with Benjamini-Hochberg correction for multiple comparisons (Table 1), linear mixed effect modeling of body weight data (Supplementary Figure 3), DESeq2 analysis with Benjamini-Hochberg adjustment of PICRUSt2 data (Supplementary Figure 2), and microbe/metabolite Spearman correlation networking (Figure 7) were performed or generated in RStudio (version 1.2.1335, RStudio Inc., Boston, MA, United States). In order to account for the factorial design of the study and to allow interaction terms while also using a non-parametric test, aligned rank transform ANOVA (Conover and Iman, 1981; Wobbrock et al., 2011) was used in lieu of a regular 2-Way ANOVA to analyze post-sleep disruption sleep (Figure 5). Mixed-effects models with Bonferroni or Benjamini-Hochberg *post hoc* testing for alpha diversity data (Supplementary Figure 1), baseline sleep data (Figure 3), during sleep-disruption sleep data (Figure 4), as well as Spearman correlations of sleep/microbiome data (Figure 6), along with generation of all other graphs/figures, were performed using GraphPad PRISM (version 9.2.0; GraphPad Inc., San Diego, CA, United States).

**TABLE 1:** Fecal bile acids across the experiment.

	Effect of diet		Effect of diet		Effect of diet		Effect of sleep disruption		Effect of social defeat	
	T2		T3		T4					
Bile Acid	*p*_*adj*_	Fold Change	*p*_*adj*_	Fold Change	*p*_*adj*_	Fold Change	*p*_*adj*_	Fold Change	*p*_*adj*_	Fold Change
Chenodeoxycholic	**0.047**	−**0.041**	0.787	0.008	0.727	0.027	**0.049**	−**0.081**	0.954	8.58E-05
Cholic	0.399	0.219	0.787	3.903	0.965	0.161	0.711	0.603	0.770	1.863
Deoxycholic	**0.047**	−**0.041**	0.787	0.008	0.727	0.027	**0.049**	−**0.081**	0.954	8.55E-05
Glycochenodeoxycholic	0.399	–0.076	0.787	0.358	0.619	0.911	0.711	–0.359	0.954	0.222
Glycocholic	0.399	0.668	0.787	0.442	0.965	–0.176	0.204	1.596	0.954	–0.101
Glycodeoxycholic	0.678	–0.012	0.787	0.128	0.619	0.601	0.578	–0.313	0.954	0.129
Glycohyocholic	0.908	0.271	0.787	2.434	0.965	0.180	0.862	0.150	0.960	0.002
Glycolithocholic	0.399	–0.512	0.787	0.178	0.965	0.122	0.711	0.770	0.770	2.245
Glycoursodeoxycholic	0.209	0.256	0.950	–0.112	0.965	–0.038	0.965	0.063	0.954	0.029
Lithocholic	**0.0004**	−**0.222**	0.787	0.130	0.965	0.041	0.197	0.197	0.954	–0.082
Muricholic	0.414	0.182	0.787	3.803	0.965	0.129	0.711	0.645	0.770	1.876
Muricholic_alpha	**0.0003**	**0.487**	0.787	–0.021	0.965	–0.074	0.711	0.113	0.954	0.056
Muricholic_beta	0.244	0.234	0.279	–0.387	0.213	–0.398	0.197	0.511	0.954	–0.106
Taurochenodeoxycholic	**0.010**	**2.739**	0.787	0.319	0.904	–0.216	0.981	0.017	0.954	0.063
Taurocholic	0.179	0.481	0.787	0.155	0.727	0.883	0.981	–0.044	0.954	0.690
Taurodeoxycholic	**0.010**	**3.12**	0.787	0.341	0.849	–0.240	0.981	0.035	0.954	0.140
Taurohyocholic	0.742	0.430	0.787	–0.073	0.213	–0.364	0.197	0.471	0.954	0.004
Taurohyodeoxycholic	0.069	1.408	0.787	–0.051	0.727	–0.187	0.197	0.385	0.954	–0.013
Taurolithocholic	0.908	0.130	0.682	3.060	0.965	–0.019	0.711	0.240	0.954	0.059
Ursodeoxycholic	0.177	–0.106	0.962	–0.020	0.965	0.067	0.981	–0.035	0.770	–0.104

*Fecal samples were collected after 4 weeks on diet (T2), at baseline sleep (T3), and at the end of the experiment (T4), and untargeted LC/MS/MS metabolomics were performed.*

*Twenty fecal bile acids were identified using purified standards.*

*The effects of diet, sleep disruption, and social defeat were assessed using Wilcoxon Rank-Sum testing (T2, T3) or linear mixed effects modeling (T4).*

*Adjusted p values (Benjamini-Hochberg correction) and fold change compared to control conditions are displayed below. Significant adjusted p values and their affiliated fold changes are indicated in bold.*

**FIGURE 2:**
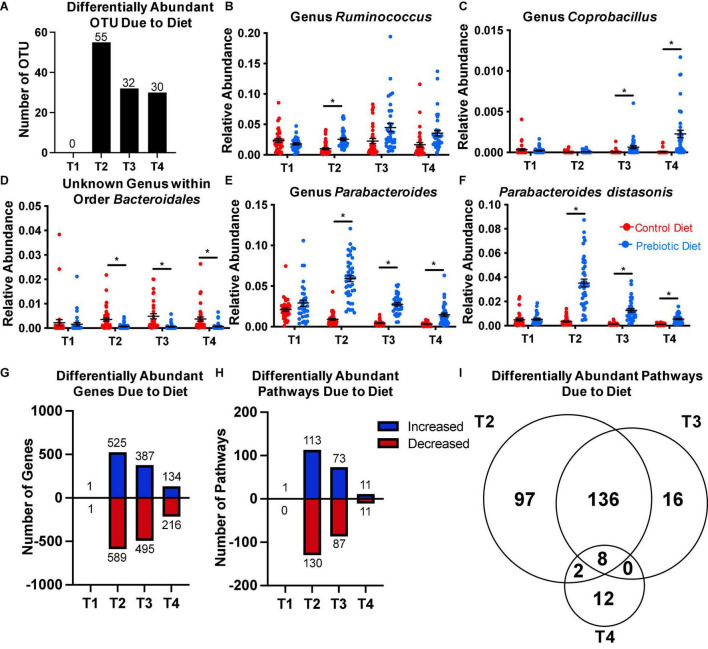
The prebiotic diet results in significant changes in the relative abundance of several taxa. Fecal samples were collected on the day of arrival to the facility (T1), after 4 weeks on diet (T2), at baseline sleep (T3), and at the end of the experiment (T4). 16S rRNA gene microbiome sequencing and analyses were performed. ANCOM was performed at each timepoint to assess differentially abundant taxa due to diet. The number of OTUs that were differentially abundant at each timepoint is reported in panel **(A)**. **(B–E)** ANCOM was then performed at the genus level, revealing four genera that were differentially abundant at ≥ 1 timepoint. **(F)** ANCOM performed at the species level confirmed *Parabacteroides distasonis* was differentially abundant at T2-T4. PICRUSt2 was then performed to predict abundance of genes and metabolic pathways based on the 16S rRNA gene data. DESEq2 was performed at each timepoint to identify differentially abundant **(G)** genes, and **(H)** pathways due to diet. **(I)** A Venn diagram depicting the number of differentially abundant pathways present at each timepoint. Data are mean ± SEM. Symbols: *differentially abundant at that timepoint *via* ANCOM. ANCOM, analysis of the composition of microbiomes; OTU, operational taxonomic unit. *n* = 33–45/group.

**FIGURE 3:**
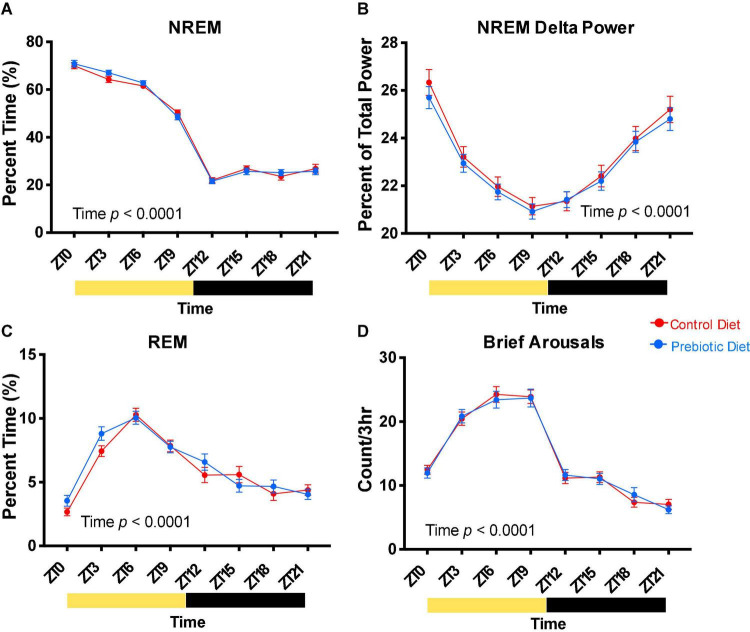
The prebiotic diet does not impact baseline sleep. Two weeks after EEG/EMG surgery, after six total weeks on diet, 24 h of baseline sleep was recorded. **(A)** NREM sleep, **(B)** NREM EEG delta power, **(C)** REM sleep, and **(D)** brief arousals are reported in 3-h bins. Yellow bars below the x axes represent times where the lights were on, while black bars represent times the lights were off. Mixed effects modeling was performed to test for effects of time, diet, and any interactions. Data are mean ± SEM. *n* = 38–40/group. EEG, electroencephalogram; NREM, non-rapid eye movement; REM, rapid eye movement; ZT, Zeitgeber time.

**FIGURE 4:**
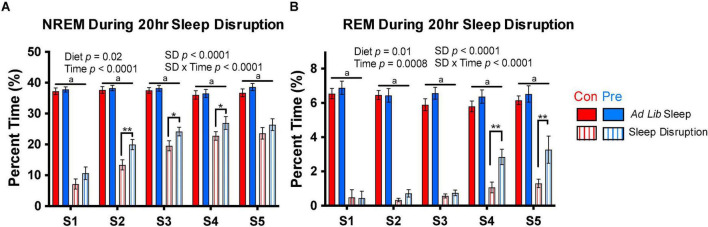
The prebiotic diet increases sleep during the sleep disruption protocol. Rats were exposed to 5 days of sleep disruption achieved by a slowly rotating bar at the bottom of the cage for 20 h per day (ZT6-ZT2). Sleep was recorded throughout this protocol, and **(A)** NREM sleep and **(B)** REM sleep during the 20-h sleep disruption periods (S1-S5) are depicted above. Mixed-effect modeling testing for an effect of timepoint, diet, sleep disruption, and interactions was performed for each measure, and significant results are reported in the figure. Data are mean ± SEM. Symbols: **q < 0.01, *q < 0.05, Fisher’s LSD test with Benjamini-Hochberg correction for multiple comparisons; *^a^q* < 0.001 for all four within-timepoint pairwise comparisons between *ad lib* sleep groups and sleep disruption groups. Con, control diet; NREM, non-rapid eye movement sleep; REM, rapid eye movement sleep; Pre, prebiotic diet. *n* = 19–24/group.

**FIGURE 5:**
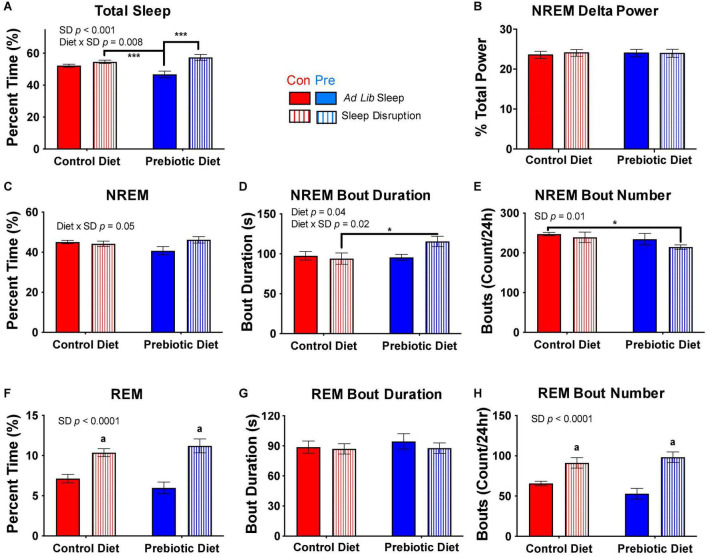
The prebiotic diet improves recovery sleep post-sleep disruption. Twenty-four hours of *ad libitum* sleep was recorded after the completion of the 5-day sleep disruption protocol. **(A)** Total sleep, **(B)** NREM EEG delta power, **(C)** NREM sleep, **(D)** median NREM bout duration, **(E)** number of NREM bouts over 24 h, **(F)** REM sleep, **(G)** median REM bout duration, and **(H)** number of REM bouts over 24 h are depicted above for the control (*ad libitum* sleep throughout) and sleep-disrupted (with no social defeat) groups. Aligned rank transform ANOVA testing for an effect of diet, sleep disruption, and interaction was performed for each measure, and significant or near significant results are reported in the figure. Data are mean ± SEM. Symbols: ***p < 0.001, *p < 0.05, Bonferroni’s *post hoc* test. *^a^ p* < 0.001 vs both control diet, *ad libitum* sleep and prebiotic diet, *ad libitum* sleep groups, Bonferroni’s *post hoc* test. Con, control diet; NREM, non-rapid eye movement sleep; Pre, prebiotic diet; REM, rapid eye movement sleep; SD, sleep disruption. *n* = 8–10/group.

**FIGURE 6:**
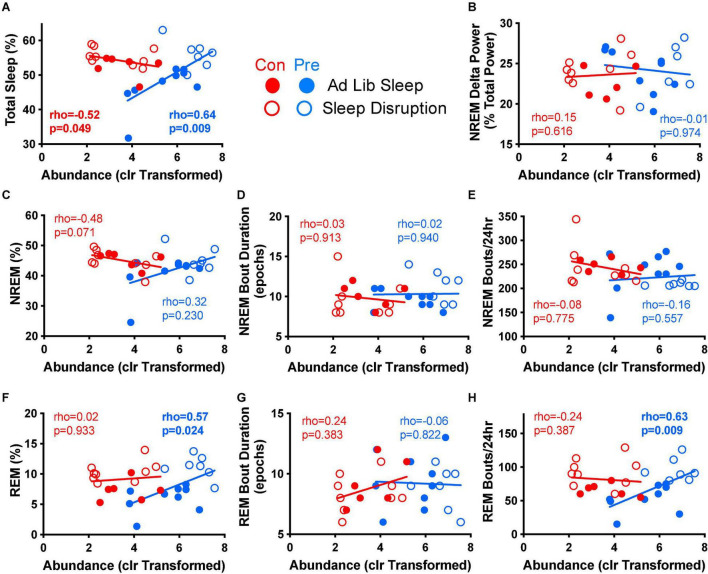
*Parabacteroides distasonis* relative abundance is associated with multiple measures of post-sleep disruption recovery sleep. Spearman’s rank-based correlation analysis between the centered log ratio transformed abundance of *P. distasonis* at the end of the experiment (T4) and various measures of recovery sleep post-sleep disruption was performed within diet group for control (no sleep disruption, no social defeat) and sleep disruption only groups. Results for **(A)** total sleep, **(B)** NREM delta power, **(C)** NREM percent, **(D)** median NREM bout duration, **(E)** NREM bouts per 24 h, **(F)** REM percent, **(G)**, median REM bout duration, and **(H)** REM bouts per 24 h are depicted above. Con, control diet; clr, centered log ratio; Pre, prebiotic diet; NREM, non-rapid-eye movement sleep, REM, rapid-eye movement sleep. *n* = 6–9/group.

**FIGURE 7:**
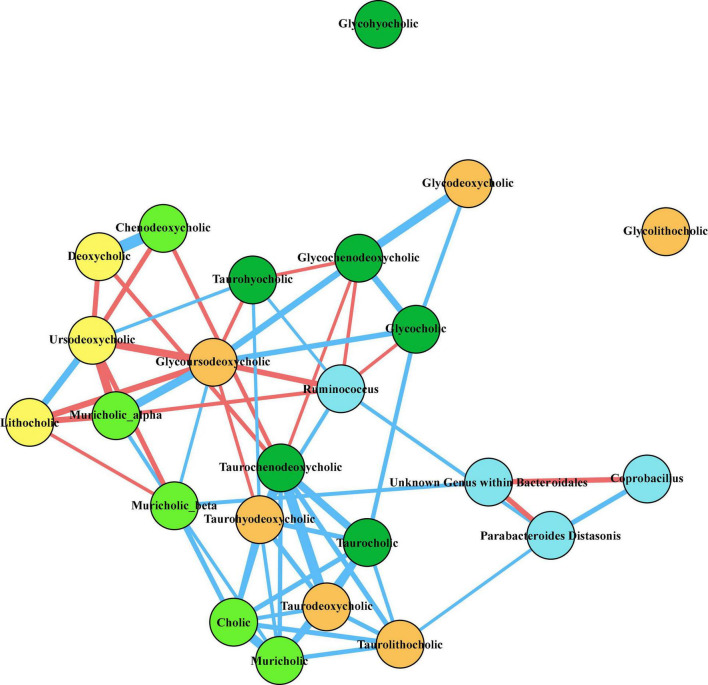
Correlation network of taxa of interest and fecal bile acids at T3. Twenty bile acids were identified from untargeted LC/MS/MS mass spectrometry of fecal samples collected at the time of baseline sleep (T3). Pairwise Spearman’s rank order correlations of bile acids and relative abundances of fecal bacteria of interest were performed across all animals, and correlations that were significant after correcting for multiple comparisons (*q* < 0.05) are depicted in the network diagram above. Nodes are colored as follows: light blue, bacteria; light green, primary bile acids; dark green, conjugated primary bile acids; yellow, secondary bile acids; orange, conjugated secondary bile acids. The thickness of the line between nodes correlates to the magnitude of Spearman’s *rho*, blue lines indicate positive *rho* values, and red lines indicate negative *rho* values.

## Results

### The Prebiotic Diet Alters the Structure and Function of the Fecal Microbiome

We first sought to characterize the effect of the prebiotic diet on the fecal microbiome over time. Rats arrived at postnatal day 23, at which time a baseline fecal sample was collected (timepoint T1). Samples were also collected at the start of the fourth week on diet (T2), the sixth week on diet (T3), and at the end of the experiment, corresponding to the seventh week on diet (T4, see Figure 1), and processed for 16S rRNA gene microbiome analysis. Unweighted and weighted UniFrac revealed significant differences in microbial community structure due to the diet at T2, T3, and T4 (Supplementary Figures 1A,B). This was accompanied by a reduction in alpha diversity in the prebiotic diet-fed rats compared to control diet-fed rats. Both Faith’s phylogenetic diversity (Supplementary Figure 1C) and the total number of observed OTU (Supplementary Figure 1D) were reduced at T2 and T3 in the prebiotic diet group compared to control, but this difference was no longer present at T4. Furthermore, Pielou’s evenness was not affected by diet condition throughout the experiment (Supplementary Figure 1E).

To characterize prebiotic diet-induced changes to the microbiome, we performed ANCOM at each timepoint to determine differentially abundant taxa. At the OTU level, ANCOM detected zero differentially abundant features at T1, 55 differentially abundant OTUs at T2, 32 differentially abundant OTUs at T3, and 30 differentially abundant OTUs at T4 (Figure 2A). Because 16S rRNA gene microbiome analysis is generally more reliable at accurate taxonomic prediction at the genus level than the species level (Gilbert et al., 2018; Knight et al., 2018), we then performed ANCOM on features that had taxonomic assignment at the genus level. We found four genera to be differentially abundant due to diet at one or more timepoints (Figures 2B–E).

*Ruminococcus* was increased at T2 in the prebiotic diet group, but not at the other timepoints (Figure 2B). The genus *Coprobacillus* was also increased due to the prebiotic diet, but only at T3 and T4 (Figure 2C). Conversely, an unknown genus within the order *Bacteroidales* was lower compared to control diet-fed rats at T2, T3, and T4 (Figure 2D). Interestingly, the genus *Parabacteroides* was greatly increased at all non-baseline timepoints, by a factor of 6.5 at T2, a factor of 7 at T3, and a factor of 5 at T4 (Figure 2E). Due to the particularly marked increase in genus *Parabacteroides* due to the diet, and the fact that there is a growing body of literature describing the role of a particular species within *Parabacteroides* (*Parabacteroides distasonis*) in host physiology (Lathrop et al., 2011; Dziarski et al., 2016; Valles-Colomer et al., 2019; Wang et al., 2019), we then performed ANCOM again at each timepoint at the species level to investigate whether *P. distasonis* was the driving factor behind the increase in genus *Parabacteroides*. Indeed, we found that *P. distasonis* was the dominant species within the genus, and that it was significantly increased at T2, T3, and T4 (Figure 2F). There were 26 OTUs that were assigned to *P. distasonis*, and the average confidence score of the assignments was 0.9755 (95% CI: 0.9459–1.005).

In order to assess whether the diet changed the function of the microbiome, we performed PICRUSt2 (Langille et al., 2013), which uses taxonomy based on 16S rRNA gene data to predict potential functional gene content of the microbiome based on reference sequences of each taxa. Then, we used DESEq2 to identify differentially abundant genes and pathways due to diet at each timepoint. We found that, using an FDR cutoff of 0.1, there were 1,114 differentially abundant genes (525 increased, 589 decreased) due to diet at T2, 882 differentially abundant genes (387 increased, 495 decreased) at T3, and 250 differentially abundant genes (134 increased, 216 decreased) at T4 (Figure 2G). Altered pathways followed a similar pattern, with 243 differentially abundant pathways (113 increased, 130 decreased) at T2, 160 differentially abundant pathways (73 increased, 87 decreased) at T3, and 22 differentially abundant pathways (11 increased, 11 decreased) at T4 (Figure 2H).

More than half of the differentially abundant pathways at T2 and T3 overlapped (144/257; Figure 2I). However, only 8 pathways were differentially abundant due to prebiotic diet at all three timepoints (Figure 2I and Supplementary Figure 2). The abundance of two pathways involved in lipopolysaccharide (LPS) synthesis were significantly increased with prebiotic diet, and two pathways relating to metabolism of exogenous molecules such as aromatic amines and nitrates were decreased due to prebiotic diet at all timepoints (Supplementary Figure 2). Furthermore, three pathways involved in sugar metabolism were altered at all time points due to the prebiotic diet (Supplementary Figure 2). Finally, abundance of a pathway involved in pyrimidine deoxyribonucleotides *de novo* biosynthesis was increased due to prebiotic diet at all three timepoints (Supplementary Figure 2). Together, these results indicate that the GOS/PDX prebiotic diet had a strong impact on the structure and function of fecal microbiome throughout the experiment, which was characterized by a 5–7-fold increase in the bacterium *P. distasonis*.

### The Prebiotic Diet Does Not Alter Baseline Sleep

Baseline sleep was assessed using EEG/EMG recording before the sleep disruption protocol (see Methods, Figure 1). We did not observe an effect of 4 weeks exposure to the prebiotic diet on NREM sleep (Figure 3A), NREM EEG delta power (Figure 3B), REM sleep (Figure 3C), or brief arousals (a measure of sleep fragmentation, Figure 3D). These measures were significantly impacted by time of day, demonstrating the circadian rhythm of these sleep parameters.

### The Prebiotic Diet Alters Sleep During and After Sleep Disruption

We measured sleep during the sleep disruption protocol to validate the efficacy of the motorized sleep disruption unit and to examine whether the prebiotic diet influenced the response to sleep restriction. As expected, we found that the sleep disruption protocol significantly reduced NREM sleep on all days of the sleep disruption protocol (Figure 4A), and nearly completely deprived rats of REM sleep, particularly on the first 3 days of the protocol (Figure 4B). The sleep disruption protocol appeared to become slightly less effective over time, as the amount of NREM and REM sleep obtained during the 20 h of sleep disruption increased over time in both groups (Figure 4). Interestingly, this process occurred more quickly in the prebiotic diet-fed rats: on days 2–4 of the sleep disruption protocol, rats in the prebiotic diet group obtained more NREM sleep during the 20-h sleep disruption period than in the control diet-fed rats (Figure 4A). The prebiotic diet-fed rats also obtained more REM sleep during the protocol than control diet-fed rats on days 4 and 5 (Figure 4B).

To assess the effect of the prebiotic diet on recovery from the 5-day sleep disruption protocol, we examined the first 24 h of recovery sleep, which began after completion of the 1-h acute social defeat stress exposure in the experimental groups or an equivalent amount of time in a clean cage in the control groups. Whereas there was no difference in total sleep due to sleep disruption in the control diet-fed rats, there was a significant increase in total sleep in the prebiotic diet-fed, sleep-disrupted rats compared to prebiotic diet-fed controls (Figure 5A). However, NREM delta power, a well-accepted measure of sleep intensity and sleep homeostatic drive (Meerlo et al., 2001; Kamphuis et al., 2015), was not changed in any group (Figure 5B). Examination of NREM sleep architecture revealed that the sleep deprived, prebiotic diet-fed rats had a trend for more NREM than non-sleep disrupted, prebiotic diet-fed rats (Figure 5C), and that this NREM sleep was more consolidated into longer bouts (Figures 5D,E). REM sleep was significantly increased during recovery in both the control diet-fed and prebiotic diet-fed groups, and this increase was due to an increase in the number of bouts without an increase in median bout length (Figures 5F–H). Thus, the prebiotic diet increased total amount of recovery sleep as compared to non-sleep disrupted animals, and promoted consolidation of recovery NREM sleep after repeated sleep disruption.

We also sought to assess the physiological impact of the sleep disruption protocol. We regularly weighed the rats throughout the experiment and found no overall effect of diet or social defeat stress on body weight (Supplementary Figure 3). Though the sleep disruption protocol did not cause a reduction in body weight, it did reduce the rate of weight gain as indicated by an overall effect of sleep disruption on body weight, as well as a sleep disruption by time interaction (Supplementary Figure 3), consistent with prior studies demonstrating increased energy expenditure in the setting of sleep restriction (Mchill and Wright, 2017). The prebiotic diet did not ameliorate this effect. We investigated whether repeated sleep disruption impacted stress-induced changes in performance in the OLM task. At the end of the sleep disruption protocol, half of the rats were exposed to 1 h of social defeat stress, while the other half were transferred to a quiet room for 1 h (see Methods). Twenty-four hours later, all animals were subjected to an OLM task in which a location index significantly above 50% is considered to indicate recognition of the moved object and retained contextual memory (see Methods). In control diet-fed rats, groups exposed to the social defeat stressor did not achieve significantly greater than 50% location index, although the mean location index for these groups was overall similar to non-stressed groups (Supplementary Figure 4). In contrast, all prebiotic diet-fed groups exhibited learning indices significantly greater than 50% (Supplementary Figure 4).

### Relative Abundance of *Parabacteroides distasonis* Correlates With Recovery Sleep

We next evaluated whether any of these prebiotic diet-induced changes to sleep were associated with the observed changes in the fecal microbiome. We focused on *P. distasonis*, as this bacterium exhibited the most pronounced increase in relative abundance in response to the prebiotic diet (Figure 2), and which has been shown in prior studies to be beneficial to host physiology (Lathrop et al., 2011; Wang et al., 2019; Thompson et al., 2021). Mean-centered log ratio (clr) transformation was then performed to convert *P. distasonis* relative abundance data to a suitable form for correlational analysis with physiological data, as relative abundances are susceptible to spurious correlations because they are compositional, such that the relative abundances within a single sample sum to one. Using clr transformation has been shown to at least partially ameliorate this problem (Gloor et al., 2017). Using this approach, we found that the clr transformed abundance of *P. distasonis* at T4 correlated positively with total sleep during the first 24 h of recovery sleep after sleep disruption in prebiotic diet-fed rats but correlated negatively with recovery sleep in control diet-fed rats (Figure 6A). *P. distasonis* also positively correlated with REM sleep and the number of REM bouts during recovery sleep (Figures 6F,H) in prebiotic diet-fed rats, both of which were altered by sleep disruption but not by the prebiotic diet (see Figure 5). *P. distasonis* did not significantly correlate with NREM delta power (Figure 6B), NREM parameters (Figures 6C–E), or median REM bout duration (Figure 6G).

### The Prebiotic Diet Alters the Fecal Bile Acid Pool

A proposed mechanism by which intestinal bacteria may influence host physiology is by generation of microbially-modified metabolites including those originating from dietary sources and host bile acids (Furusawa et al., 2013; De Vadder et al., 2014; Kuipers et al., 2014; Govindarajan et al., 2016; Stilling et al., 2016; Yanguas-Casas et al., 2017). Interestingly, a recent study of *P. distasonis* demonstrated that it exerts metabolic benefits in part *via* secondary bile acid production (Wang et al., 2019). We therefore evaluated the impact of the prebiotic diet on the fecal metabolome, and specifically on the bile acid pool, by performing untargeted LC/MS/MS mass spectrometry on fecal samples taken throughout the experimental protocol. We identified 20 different unconjugated and conjugated primary and secondary bile acids within the final feature tables using purified standards and investigated whether the diet altered levels of these bile acids at T2, T3, or T4 (Table 1). We found that at T2 (4 weeks on diet) there were six bile acids that were significantly altered due to diet after correcting for multiple comparisons. The primary bile acid muricholic acid was decreased, while another primary bile acid chenodeoxycholic acid was increased (Table 1). The conjugated primary bile acid taurochenodeoxycholic acid was also increased. Secondary bile acids deoxycholic acid and lithocholic acid were lower in the prebiotic diet group, and the conjugated secondary bile acid taurodeoxycholic acid was significantly increased in the prebiotic group. A number of these changes are consistent with our prior observations from independent experiments at a separate facility (Thompson et al., 2021). These diet effects were no longer present at T3 or T4 (Table 1). There was an overall effect of sleep disruption on the primary bile acid chenodeoxycholic acid and the secondary bile acid deoxycholic acid, but no other bile acids were affected by sleep disruption, and none of the 20 identified bile acids were impacted by social defeat stress exposure (Table 1).

To investigate whether the microbes we found to be most affected by the prebiotic diet were related to the fecal bile acid pool, we performed pairwise Spearman correlations of clr transformed abundances of *P. distasonsis, Ruminococcus, Coprobacillus*, and the unknown order within *Bacteroidales* (see Figure 2) with the normalized abundances of the 20 identified bile acids at T3 (time of baseline sleep). The resultant network (Figure 7) of significant (*q* < 0.05) correlations revealed a tightly covarying network of primary and secondary bile acids, such that *P. distasonis*, *Ruminococcus*, and the unknown order within *Bacteroidales* all correlated with at least one bile acid, thus integrating the observed microbial changes with the detected alterations in the fecal bile acid pool.

## Discussion

In this study, we tested the hypothesis that dietary supplementation with the prebiotics GOS and PDX improves sleep in response to repeated sleep restriction in adult male rats. We demonstrate a significant impact on our primary outcome measure, demonstrating that the prebiotic diet leads to significant increases in sleep during both the sleep restriction and recovery sleep phases of the experimental protocol. In addition, as expected, the prebiotic diet exerts a significant and stable effect on the structure and predicted function of the microbiome (Figure 2). The impact on the microbiome is most prominent at time point 2 (T2), which occurred prior to the sleep restriction and acute stress exposure portions of the protocol and represents the effect of 4 weeks of the dietary intervention.

Alpha diversity was decreased in the animals exposed to the prebiotic diet, particularly at T2 and T3 (Supplementary Figure 1). Increases in diversity are generally thought beneficial as decreased diversity has been associated with adverse physiologic states such as stress exposure (Bharwani et al., 2016; Thompson et al., 2016) and diseases such as inflammatory bowel disease (Nishino et al., 2018), whereas increased diversity is presumed to represent a healthier and more resilient resident microbial ecosystem. However, quantification of taxonomic representation is agnostic to the physiological function of the taxa that are tallied. Thus, it is possible that despite an overall reduction in diversity, there is a relative increase in beneficial bacteria accompanied by a larger decrease in detrimental and/or neutral taxa, leading to a net positive physiologic status change despite a lower total number of taxa. For example, a large “bloom” of a beneficial species (such as *P. distasonis*, potentially) induced by the prebiotic diet may suppress other bacteria leading to an overall decrease in diversity. Recent published examples are compatible with this hypothesis that a net positive physiologic change can occur in the setting of decreased overall diversity: in a study comparing healthy individuals to patients with major depressive disorder, the healthy control subjects exhibited a decrease in alpha diversity compared to those with depression (Jiang et al., 2015); and in a study examining infants, individuals with a lower alpha diversity at 1 year of age had better performance on a validated learning scale at 2 years of age compared to those with greater alpha diversity at age 1 (Carlson et al., 2018).

Looking beyond the structural changes to the microbiome, further analysis demonstrates that multiple genes and pathways are significantly altered by the prebiotic diet (Figure 2). Of all pathways affected, a subset of eight are consistently changed in the same direction at each time point. These eight include pathways involved in LPS synthesis and the metabolism of carbohydrates and exogenous molecules (Supplementary Figure 2), suggesting that a major functional impact of the prebiotic diet is the regulation of lipopolysaccharide synthesis and of specific microbial metabolic pathways, which together may contribute to the observed microbial structural as well as the host physiologic changes induced by the prebiotic diet.

The largest dietary effects on the microbiome were seen at T2, prior to the sleep restriction and stress exposure components of the protocol, with overall lower numbers of differentially affected taxa, genes, and pathways at T3 and T4 (Figure 2). The reason for this pattern of changes is unclear, but may be related to an evolving microbial ecosystem in flux in response to prebiotic diet exposure. At T2, most notable among the many significant changes is the dramatic rise in *P. distasonis* relative abundance. We are aware of the limited resolution of taxonomic assignment using 16S rRNA gene analysis, but we are confident this taxonomic classification is accurate. Over time, the total number of significantly altered taxa, genes, and pathways may decrease as the microbial ecosystem moves toward a new “set point,” perhaps explaining the fewer differential effects noted at T3 and T4. Future work may help better characterize the long-term complex microbial and metagenomic changes induced by the prebiotic diet, as well as the time course and dynamics of these changes.

The prebiotic diet does not impact baseline sleep (Figure 3) but does lead to significant increases in NREM and REM sleep during the sleep restriction protocol (Figure 4) and total sleep, NREM sleep, and NREM bout duration during the sleep recovery period (Figure 5). Thus, while the prebiotic diet did not impact sleep at baseline, it enabled rats to get more sleep *during* the sleep disruption protocol. Taken together, these results suggest that the prebiotic diet enables animals to better handle the physiologic challenge of experimental sleep restriction by improving their ability to obtain sleep during active sleep restriction as well as during the following recovery period.

The relative abundance of *P. distasonis* was found to positively correlate with several sleep parameters in prebiotic diet-fed rats, such as total sleep and REM sleep as well as total bouts of REM sleep (Figure 6). This suggests that *P. distasonis* may exert a particularly important role in mediating the sleep-promoting effects of the prebiotic diet. *Parabacteroides distasonis* is a commensal gram-negative bacterium that has previously been shown to exert immunomodulating properties in the intestine *via* induction of T_*regs*_ (Lathrop et al., 2011), ameliorate metabolic dysfunction in a genetic model of obesity and in mice fed a high-fat diet (Wang et al., 2019), help promote quicker re-entrainment in response to circadian rhythm disruption (Thompson et al., 2021), compensate for inadequate dietary protein intake in a mouse model of malnourishment (Martin et al., 2021), and contribute to resilience against metabolic, behavioral, and neurocognitive responses to chronic restraint stress (Deng et al., 2021).

In contrast to these beneficial effects associated with *P. distasonis*, other studies have found adverse consequences, such as increased susceptibility to dextran sodium sulfate-induced colitis in mice treated with *P. distasonis via* oral gavage (Dziarski et al., 2016), exacerbation of disease phenotype in a mouse genetic model of amyotrophic lateral sclerosis when antibiotic-treated mice were supplemented with *P. distasonis* (Blacher et al., 2019), and depressive-like behavior in a genetically-induced mouse model of Crohn’s disease-like ileitis (Gomez-Nguyen et al., 2021). These disparate findings suggest that the impact of *P. distasonis* may be context-dependent, strain-dependent, related to the underlying physiologic state of the host, and/or dependent on the way in which *P. distasonis* is augmented. For example, dietary prebiotic supplementation to increase relative abundance in otherwise healthy mice may yield different effects than those achieved by oral gavage or in the setting of an otherwise healthy intestine as opposed to an inflamed intestine or an intestine characterized by an altered microbial ecosystem depleted by antibiotic exposure.

There are multiple possible mechanisms by which changes to the intestinal microbiome may impact host physiologic processes, including physical host-microbe interactions, bile acid modification, production of metabolites, modulation of signaling pathways, and immune regulation (Lavelle and Sokol, 2020; Sipe et al., 2020; Agus et al., 2021). A particularly relevant possibility is that changes to bile acids induced by secondary microbial metabolism may then contribute to systemic physiologic effects in the host. Our analysis of fecal bile acids demonstrates consistent directional relationships between bacterial changes induced by the prebiotic diet and alterations in the fecal bile acid profiles, suggesting that, given the known functional capacity of the involved bacterial taxa, the metagenomic changes to the microbial ecosystem in response to the prebiotic diet are a reasonable explanation for the observed bile acid changes.

There are several limitations to our study that are important to consider. The study was performed on adult male rats, so the applicability to other model systems and to humans may be limited. Our experimental protocol incorporated exposures to dietary intervention, sleep restriction, and acute social defeat stress. Thus, the observed significant effects may be particular to the types of experimental manipulation and/or sequence of exposures, with different effects possible in other models or experimental contexts. Similarly, our outcome measurements related to the microbiome, fecal metabolome, and sleep, are limited to specific timepoints. These static “snapshots” of time during the protocol may not completely capture or characterize evolving physiologic processes related to the regulation of sleep and the gut microbial ecosystem that may not have yet reached equilibrium at the time of measurement or sample collection. The links between sleep parameters and changes to the microbiota and fecal metabolites described here are inherently correlational in nature, thus they are unable to reveal underlying causal relationships or mechanisms of action. Statistical techniques have been employed to infer information about the relationships described, but the analyses used here are ultimately limited, with the capability of identifying hypotheses and future experiments necessary to parse the biological mechanisms driving the observed effects.

Despite these limitations, our finding of a prebiotic diet capable of enhancing resilience to sleep disruption offers a unique opportunity to combat the common and adverse consequences of sleep deprivation by utilizing a dietary strategy to promote sleep *via* enhancement of the microbiome. Future work should delineate the causal relationships and underlying mechanisms driving the effects of the prebiotic diet on the microbiome, the fecal metabolome, and the regulation of sleep, in order to identify more precise therapeutic targets. In addition, further work should examine the role of prebiotic diet supplementation in other model systems and in other physiologically challenging states associated with insufficient or poor sleep, to investigate the replicability and generalizability of the prebiotic diet’s sleep-promoting effects.

## Data Availability Statement

The datasets presented in this study can be found in online repositories. The names of the repository/repositories and accession number(s) can be found in the article/Supplementary Material.

## Ethics Statement

The animal study was reviewed and approved by Northwestern University Institutional Animal Care and Use Committee.

## Author Contributions

SB and KS wrote the first draft of the manuscript and incorporated contributions from all co-authors into the final draft, which was approved by all authors prior to submission. SB, CO, PJ, AG, and FV carried out studies and collected data. SB, KS, RT, AG, FV, and PJ analyzed the data. SB, KS, RT, AG, FV, PJ, CL, PD, RK, KW, MF, FT, and MV interpreted the data. CL, PD, RK, KW, MF, FT, and MV designed the studies and obtained funding. All authors contributed to the article and approved the submitted version.

## Conflict of Interest

KW reports research support/donated materials from DuPont Nutrition & Biosciences; Grain Processing Corporation; and Friesland Campina Innovation Centre. Financial relationships: consulting with or without receiving fees and/or serving on the advisory boards for Circadian Therapeutics, Ltd., Circadian Biotherapies, Inc., Philips Respironics, and the United States Army Medical Research and Materiel Command – Walter Reed Army Institute of Research. The remaining authors declare that the research was conducted in the absence of any commercial or financial relationships that could be construed as a potential conflict of interest.

## Publisher’s Note

All claims expressed in this article are solely those of the authors and do not necessarily represent those of their affiliated organizations, or those of the publisher, the editors and the reviewers. Any product that may be evaluated in this article, or claim that may be made by its manufacturer, is not guaranteed or endorsed by the publisher.

## Footnotes

1
https://github.com/picrust/picrust2

## Supplementary Material

Supplementary Figure 1 The Prebiotic Diet Causes Changes to Fecal Microbiome Beta and Alpha Diversity. Fecal samples were collected on the day of arrival to the facility (T1), after 4 weeks on diet (T2), at baseline sleep (T3), and at the end of the experiment (T4). 16S rRNA gene microbiome sequencing and analyses were performed. PERMANOVA testing for an effect of diet was performed at each timepoint, and PCoA depicting **(A)** unweighted UniFrac and **(B)** weighted UniFrac analysis of beta diversity, with one axis representing timepoint, are reported. Alpha diversity was measured at each timepoint using **(C)** Faith’s phylogenetic diversity index, **(D)** the total number of OTU, and **(E)** Pielou evenness metric. Mixed-effect modeling testing for an effect of timepoint, diet, and interactions was performed for each dependent variable, and significant results are reported in the figure. Data are mean ± SEM. Symbols: **(A,B)** ^***^*p* < 0.001, ^**^*p* < 0.01, PERMANOVA; **(C–E)** ^**^*p* < 0.01, **p* < 0.05, Bonferroni *post hoc*. Abbreviations: OTU, operational taxonomic unit; PC, principal coordinate. *n* = 33–45/group.

Supplementary Figure 2 Predicted Microbial Metabolic Pathways Altered by the Prebiotic Diet. Fecal samples were collected after 4 weeks on diet (T2), at baseline sleep (T3), and at the end of the experiment (T4). 16S rRNA gene microbiome sequencing and analyses were performed. PICRUSt2 was performed on the 16S rRNA gene microbiome data to predict genetic content. DESEq2 was then performed at each timepoint to identify predicted pathways that were differentially abundant due to diet. The above reports the pathway ID, description, fold change, and direction of change of the 8 pathways that were significantly altered by diet at T2, T3, and T4.

Supplementary Figure 3 The Prebiotic Diet Does Not Prevent Sleep Disruption-Induced Changes in Body Weight. Animals were weighed throughout the experiment. Day 0 indicates the start of baseline sleep recording. Results of linear mixed effect modeling investigating overall effects and interactions with time are depicted above. Data represent mean ± SEM. *n* = 9–12/group.

Supplementary Figure 4 The Prebiotic Diet Prevents Social Defeat-Induced Loss of Object Location Memory. Immediately after the end of the last sleep disruption period, half of the rats were exposed to 1 h of social defeat while the other half were exposed to a clean cage for an equivalent time period. Twenty-four hours later, object location memory was assessed in all rats. Location indices above 50% indicate retained contextual memory and learning. Data represent mean ± SEM. Symbols: ^*p* < 0.05, one sample Wilcoxon Rank-Sum test vs. 50%. Abbreviations: Con, control diet; Pre, prebiotic diet. *n* = 9–12/group.

## Abbreviations

ANCOM: analysis of composition of microbiomes
BL: baseline
clr: centered log ratio
Con: control diet
EEG: electroencephalography
EMG: electromyography
GOS: galactooligosaccharides
LPS: lipopolysaccharide
NREM: non-rapid eye movement
OLM: object location memory
P: postnatal day
PC: principal coordinate
PCoA: principal coordinate analysis
PDX: polydextrose
PERMANOVA: permutational multivariate analysis of variance
Pre: prebiotic diet
REM: rapid eye movement
SD1-5: 24-h sleep disruption day 1-5
S1-5: 20-h sleep disruption window
ZT: Zeitgeber time.
