# Supplementary figures and images for: A Prebiotic Diet Alters the Fecal Microbiome and Improves Sleep in Response to Sleep Disruption in Rats
Source: Front Neurosci. 2022 May 24;16:889211. doi: 10.3389/fnins.2022.889211 (PMC9172596; doi:10.3389/fnins.2022.889211)

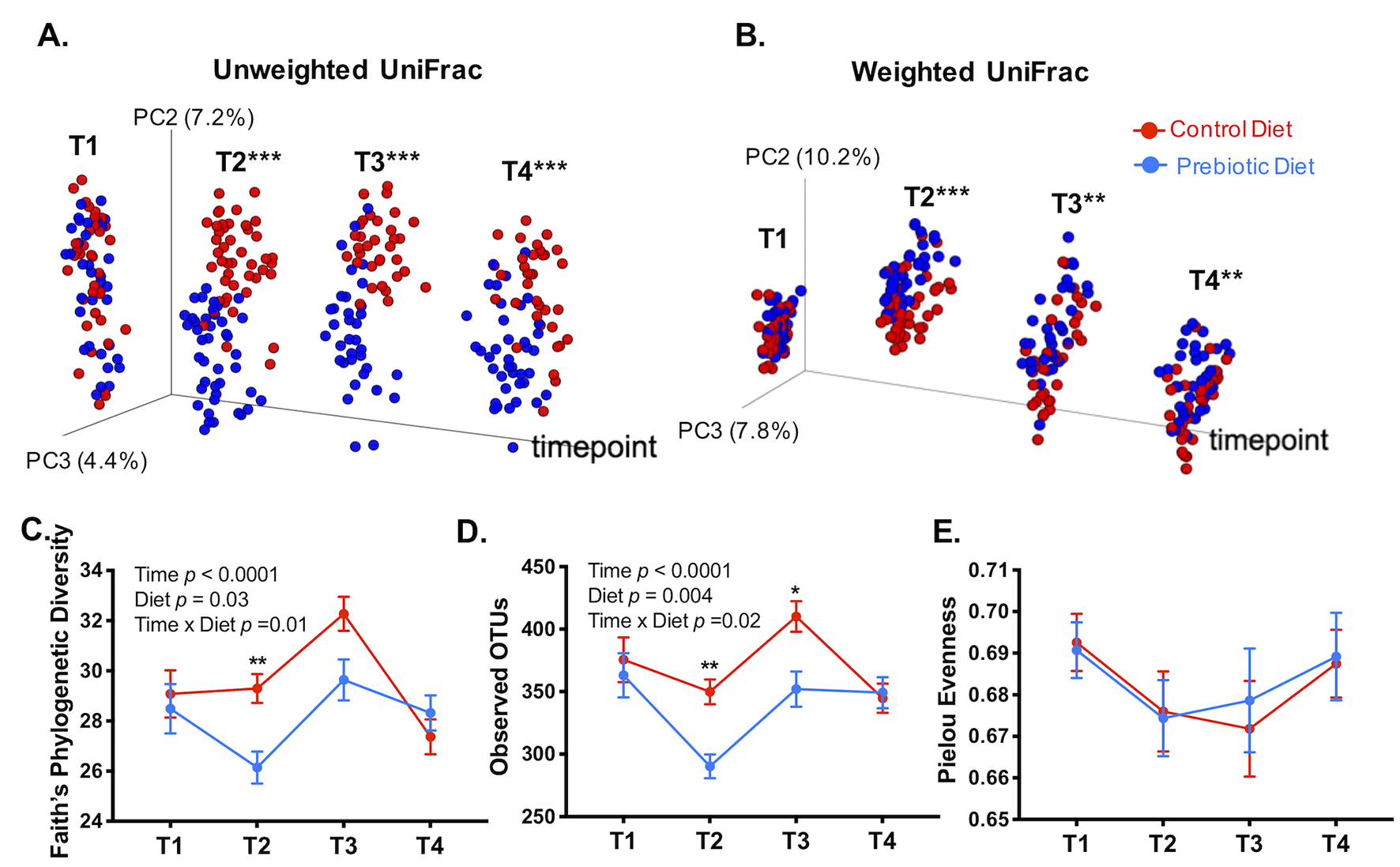

Supplement: Supplementary Figure 1 — The Prebiotic Diet Causes Changes to Fecal Microbiome Beta and Alpha Diversity. Fecal samples were collected on the day of arrival to the facility (T1), after 4 weeks on diet (T2), at baseline sleep (T3), and at the end of the experiment (T4). 16S rRNA gene microbiome sequencing and analyses were performed. PERMANOVA testing for an effect of diet was performed at each timepoint, and PCoA depicting (A) unweighted UniFrac and (B) weighted UniFrac analysis of beta diversity, with one axis representing timepoint, are reported. Alpha diversity was measured at each timepoint using (C) Faith’s phylogenetic diversity index, (D) the total number of OTU, and (E) Pielou evenness metric. Mixed-effect modeling testing for an effect of timepoint, diet, and interactions was performed for each dependent variable, and significant results are reported in the figure. Data are mean ± SEM. Symbols: (A,B) ***p < 0.001, **p < 0.01, PERMANOVA; (C–E) **p < 0.01, *p < 0.05, Bonferroni post hoc. Abbreviations: OTU, operational taxonomic unit; PC, principal coordinate. n = 33–45/group. [file Image_1.TIFF]

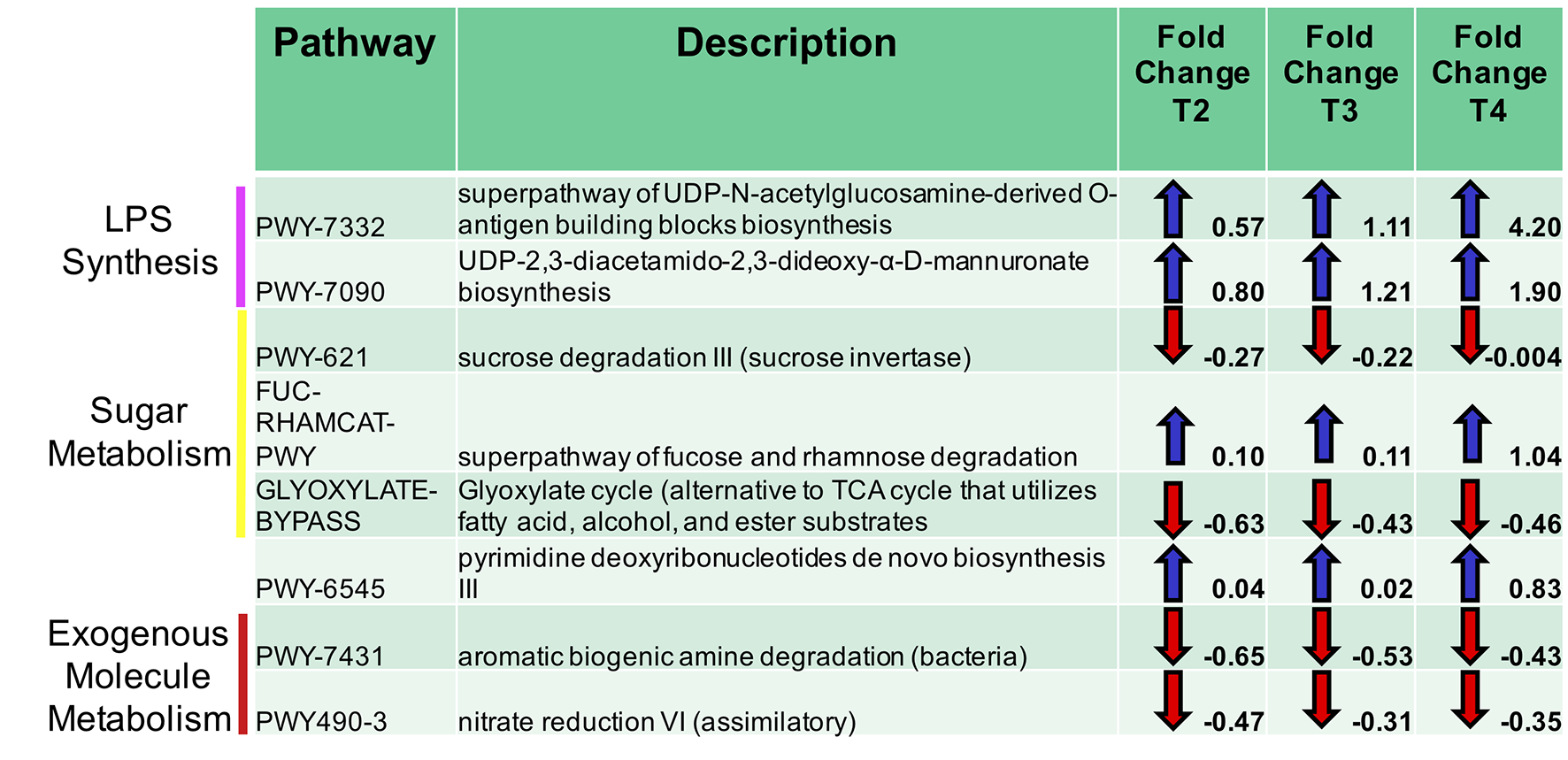

Supplement: Supplementary Figure 2 — Predicted Microbial Metabolic Pathways Altered by the Prebiotic Diet. Fecal samples were collected after 4 weeks on diet (T2), at baseline sleep (T3), and at the end of the experiment (T4). 16S rRNA gene microbiome sequencing and analyses were performed. PICRUSt2 was performed on the 16S rRNA gene microbiome data to predict genetic content. DESEq2 was then performed at each timepoint to identify predicted pathways that were differentially abundant due to diet. The above reports the pathway ID, description, fold change, and direction of change of the 8 pathways that were significantly altered by diet at T2, T3, and T4. [file Image_2.TIFF]

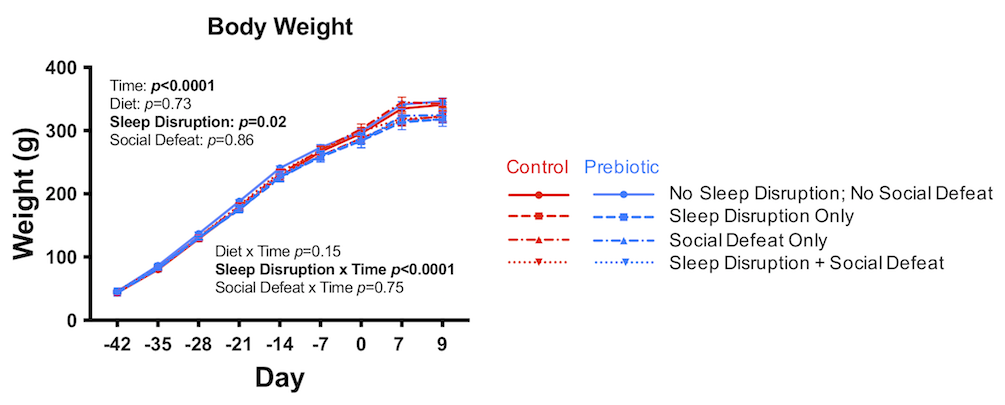

Supplement: Supplementary Figure 3 — The Prebiotic Diet Does Not Prevent Sleep Disruption-Induced Changes in Body Weight. Animals were weighed throughout the experiment. Day 0 indicates the start of baseline sleep recording. Results of linear mixed effect modeling investigating overall effects and interactions with time are depicted above. Data represent mean ± SEM. n = 9–12/group. [file Image_3.TIFF]

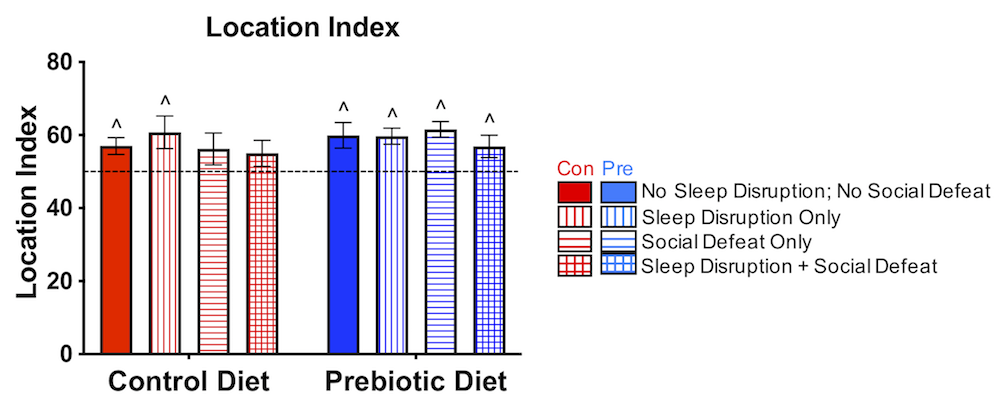

Supplement: Supplementary Figure 4 — The Prebiotic Diet Prevents Social Defeat-Induced Loss of Object Location Memory. Immediately after the end of the last sleep disruption period, half of the rats were exposed to 1 h of social defeat while the other half were exposed to a clean cage for an equivalent time period. Twenty-four hours later, object location memory was assessed in all rats. Location indices above 50% indicate retained contextual memory and learning. Data represent mean ± SEM. Symbols: ^p < 0.05, one sample Wilcoxon Rank-Sum test vs. 50%. Abbreviations: Con, control diet; Pre, prebiotic diet. n = 9–12/group. [file Image_4.TIFF]
